# New insights regarding tissue Se and Hg interactions on oxidative stress from plasma IsoP and IsoF measures in the Canadian Inuit population

**DOI:** 10.1194/jlr.M033068

**Published:** 2013-07

**Authors:** Dalal Alkazemi, Grace M. Egeland, L. Jackson Roberts, Hing M. Chan, Stan Kubow

**Affiliations:** * School of Dietetics and Human Nutrition, McGill University, Ste-Anne-de-Bellevue, QC H9X 3V9, Canada; † Centre for Indigenous Peoples’ Nutrition and Environment (CINE), McGill University, Ste-Anne-de-Bellevue, QC H9X 3V9, Canada; § Norwegian Institute of Public Health and Department of Global Public Health and Primary Care, Faculty of Medicine and Dentistry, University of Bergen, N-5018 Bergen, Norway; ** Department of Pharmacology and Medicine, Vanderbilt University, Nashville, TN 37232; †† Center for Advanced Research in Environmental Genomics, University of Ottawa, Ottawa, ON K1N 6N5, Canada

**Keywords:** isoprostane, isofuran, C-reactive protein, mercury, selenium

## Abstract

Despite animal and in vitro studies demonstrating pro-oxidative effects of Hg, previous human work showed no relationship between tissue Hg and plasma levels of F_2_-isoprostanes (IsoPs), a whole-body oxidative stress marker. We hypothesized that another IsoP species, isofurans (IsoFs), was a more sensitive indicator of Hg-mediated oxidative stress, which can be modified by tissue Se status. A cross-sectional study was carried out involving individuals from a random subset (n = 233) of Inuit adults from a population-based survey (n = 2,595) of 36 Canadian Arctic Inuit communities to assess the relationships of plasma IsoPs to Se and Hg status indicators. F_2_-IsoPs were inversely correlated with blood Se (*r* = −0.186, *P* = 0.005) and toenail Se (*r* = −0.146, *P* = 0.044), but not correlated with Hg. IsoFs were inversely correlated with blood Se (*r* = −0.164, *P* = 0.014) and positively correlated with Hg (*r* = 0.228, *P* < 0.001) and Hg:Se (*r* = 0.340, *P* < 0.001). The strength of the correlations remained unchanged after multivariate adjustments. Multivariate analysis showed that F_2_-IsoPs were not positively associated with Hg but with Hg:Se (β = 0.148, *P* = 0.021). We conclude that Se and Hg status and their interactions are important factors modulating F_2_-IsoP and IsoF levels such that the Inuit may be protected from Hg-induced oxidative stress because of their high Se status.

Methylmercury (MeHg) is postulated to increase cardiovascular risk via several pathways, including direct cardiovascular effects (such as decreased heart rate variability, increased blood pressure, and reduced myocardial contractile force) and systemic effects involving blood coagulation, inhibition of endothelial cell migration, and greater oxidative stress via the promotion of free radicals and reactive oxygen species, lipid peroxidation, and inhibition of antioxidant systems (1–3). Epidemiological studies of health outcomes such as hypertension and myocardial infarction are conflicting (4–9), perhaps owing to differences in coexposures to beneficial nutrients such as long-chain n-3 fatty acids and Se. Direct antagonistic effects of MeHg on Se-dependent antioxidant enzymes have been noted (10). Additionally, sequestration of Se caused by Hg-Se adducts can lead to a functional deficit of tissue Se caused by decreased availability of Se for incorporation into selenoproteins involved in antioxidant defense (11). MeHg-induced oxidative stress has also been associated with depressed tissue levels of sulfhydryl-dependent antioxidant proteins and the reduced form of glutathione (GSH) (12). Increased dietary Se, however, can counteract the sequestration of Se by Hg-Se adducts (13) and so maintain normal selenoenzyme activities (14).

Animal studies have generally indicated that Se intake mitigates against dietary MeHg toxicity, which appears to be related to both the absolute and relative amounts of MeHg and Se present in the diet (14, 15). The examination of the diets and tissues of MeHg-exposed animals has shown that Se:Hg molar ratios above one are protective against adverse effects associated with MeHg exposure (14). Human studies, however, have shown mixed results regarding associations between tissue Se status and adverse outcomes from MeHg exposure and so their interactions remain unclear (1, 6).

Inuit have high exposures to Se and n-3 polyunsaturated fatty acids (PUFAs) compared with Caucasians (16, 17) as the seafood-based Inuit traditional diet has a rich content of Se and n-3 PUFAs, which are considered protective against cardiovascular disease (CVD) (16, 18). Additionally, Inuit appear to be protected against oxidative stress-mediated complications associated with hyperlipidemia as shown by the absence of elevated oxidized LDL in association with atherogenic blood lipid values (17, 18). The Inuit diet, however, is associated with MeHg exposure through the consumption of traditional food. Elevated exposure of MeHg among Inuit may be associated with cardiovascular risk as it was positively and independently associated with systolic blood pressure (SBP) and pulse pressure (19); which may indicate interference with the normal functioning of the cardiovascular system (20–22).

It is unclear, however, whether the high Se intake from the traditional Inuit diet protects against MeHg-mediated oxidative stress. Inuit subjects from Nunavik were recently shown to have elevated levels of plasma lipophilic antioxidants such as α-tocopherol, ubiquinone-10, and coenzyme Q_10_ in comparison with healthy Caucasian controls, but no association between these plasma biomarkers was seen with MeHg or other markers of traditional food intake (23). Belanger et al. (17) suggested that an unusually elevated ratio of ubiquinone-10 to total CoQ_10_ in Inuit plasma was reflective of oxidative stress and that the high levels of plasma antioxidant components could reflect an adaptive response to an oxidative stress of undetermined origin. Assessment of antioxidant-related plasma components, however, may have limited sensitivity to assess the interrelationship between dietary MeHg exposure and tissue Se on oxidative stress status.

In the present study, we evaluate the associations of MeHg and Se exposures with F_2_-isoprostanes (IsoPs), which is one of the most reliable approaches for in vivo assessment of whole body oxidative stress (24). While an inverse association between blood Se concentrations and F_2_-IsoPs has been noted in Finland (25), the relationship between tissue Se status and dietary MeHg exposure has not been studied with respect to F_2_-IsoP levels. Further, we evaluate the ratio of isofurans (IsoFs) to F2-IsoPs to obtain a more comprehensive view of possible oxidative injury. IsoFs are products of lipid peroxidation with a substituted tetrahydrofuran ring (26). The formation of F_2_-IsoPs and IsoFs is differentially regulated by oxygen tension; the formation of F_2_-IsoPs is favored by low oxygen tension whereas the formation of IsoFs is favored by elevated oxygen tension that can occur in settings of mitochondrial dysfunction (26).

In a subsample of the International Polar Year (IPY) Inuit Health Survey study population, we showed that IsoFs and F_2_-IsoPs were positively associated with pro-inflammatory C-reactive protein (CRP), after adjustments for covariates (27). Oxidative stress and inflammation are considered contributing components to the progression of atherosclerosis (28) and, thereby putative mediators of MeHg's adverse cardiovascular effects. In the current study the association between F_2_-IsoPs and IsoFs was examined with respect to tissues reflecting short-term (blood) Hg and Se and long-term Se (toenail) intake of MeHg and Se and their relationship with CVD risk factors. An additional aim was to examine how much of the variability in plasma F_2_-IsoP and IsoF levels can be explained by Hg body burden, the Hg-Se interaction, and cardiometabolic health outcomes. We hypothesized that plasma levels of IsoPs and IsoFs would associate with Hg exposure and that enhanced tissue Se status would protect against MeHg-related oxidative stress.

## SUBJECTS AND METHODS

### Subject recruitment

The current study is based upon a random subsample of participants of a population-based IPY Inuit Health Survey, details of which are available elsewhere (29). In brief, a cross-sectional survey was conducted in the summer and fall 2007 and 2008 for 33 coastal communities and for three noncoastal communities representing all communities in the Inuvialuit Settlement Region (ISR, Northwest Territories) and the Nunavut and Nunatsiavut Region (Northern Labrador). Trained interviewers and nursing staff collected information on subjects’ dietary habits, physical activity, psychosocial factors, medical history, blood pressure, anthropometric indices, fasting lipids, and various clinical indices. Fasting blood samples were prepared and stored at −80°C for future analyses. Territorial research licenses were obtained and the Ethical Review Board of the McGill University Faculty of Medicine approved the study. Informed consent was obtained from all participants prior to enrollment.

### Anthropometric, physiologic measures and definitions for categorical variables

Height, weight, waist circumference (WC), and blood pressure were measured during clinical session, and were performed by a trained research nurse according to a standard protocol as previously reported (30). A body mass index (BMI) of 25.0–29.9 kg/m^2^ was considered overweight and a BMI of 30 kg/m^2^ or greater was considered obese.

### Laboratory methods

Fasting serum total cholesterol, high density lipoprotein cholesterol (HDL-C), and triglycerides were determined using enzymatic colorimetric tests and low density lipoprotein cholesterol (LDL-C) was calculated by Nutrasource Diagnostics, Guelph, ON (Life Laboratories, Gamma Dynacare). Serum high sensitivity CRP concentration was determined using immunoturbidimetric assay with SYNCHRON® high sensitivity CRPH reagent in conjunction with SYNCHRON® Systems CAL 5 Plus (Beckman Coulter Inc., Fullerton, CA) in the Centre for Indigenous Peoples’ Nutrition and Environment (CINE) at McGill University.

### Assessment of Hg and Se exposure

Analyses for Hg and Se were performed at the Laboratoire de Toxicologie, Institut national de santé publique, Quebéc, on whole blood. Briefly, blood samples were diluted in a basic solution containing octylphenol ethoxylate and ammonia, which was followed by inductively coupled plasma mass spectrometry. Matrix-matched calibration was performed using blood from a nonexposed individual. Toenails incorporate Se and may reflect dietary intake over the past year (31). Toenail Se analysis was performed at the laboratories of CINE and the Department of Natural Resource Sciences at McGill University. Briefly, samples were cleaned in acetone and distilled water, then digested in concentrated nitric acid at 110°C for 4 h. Digests were dried at 160°C and reconstituted in 2% nitric acid. Se concentrations were measured using a Varian model ICP 820-MS with a collision reactor interface.

### Plasma analysis of F_2_-IsoPs and IsoFs

Plasma samples were prepared from fasting blood samples and stored at −80°C until time of analysis. Purification, derivatization, and analysis of F_2_-IsoPs and IsoFs by stable isotope dilution gas chromatography-negative ion chemical ionization-mass spectrometry (GC-NICI-MS) were performed as previously described (24). An Agilent 5973 mass spectrometer coupled to an Agilent 6890N gas chromatograph using a 15 mDB 1701 GC column was utilized with an inlet temperature of 260°C. The helium carrier gas flow rate was 2 ml/min. For sample injection, the GC oven was programmed to run from 190 to 300°C at 20°C/min for 9 min. Selective ion GC-NICI-MS monitoring was 569 *m/z* for F_2_-IsoPs, 585 *m/z* for IsoFs, and 573 *m/z* for the internal standard [^2^H_4_]15-F2t-IsoP. Plasma values are expressed in pg/ml. The precision of the assay is ±6% and the accuracy is 96%.

### Statistical analysis

Anthropometric, clinical, and biochemical measures for all subjects were reported as mean ± SD, or for skewed variables, as geometric mean and 95% confidence intervals (CIs) from log-transformed variables. All variables were evaluated as continuous data in statistical analyses. Plasma F_2_-IsoPs, IsoFs, and whole blood Hg, Se, and CRP were skewed and log-transformed before analysis. Both IsoFs:F_2_-IsoPs Hg:Se ratios were calculated using the log-transformed data for F_2_-IsoPs, IsoFs, Hg, and Se respectively. The prevalence of preexisting medical conditions was determined based on self-reported information through interviews with nurses. Participants provided their medications and supplements to nurses for correct documentation. Correlation analysis was performed using Pearson's correlation analysis to assess the relationship between plasma concentrations of measures of oxidative stress (F_2_-IsoPs, IsoFs) and parameters of interest (Se, Hg, Hg:Se ratio, and toenail Se), in addition to their relationship to other study parameters including obesity measures and cardiovascular risk factors. Baseline characteristics were compared between Se tertiles using ANOVA and Boneferroni's post hoc test when a significant group effect was observed. All *P* values were two tailed, and *P* < 0.05 was considered significant for all tests performed. General linear models were used to calculate adjusted means of plasma IsoPs adjusting for important covariates including age, WC, and sex. To estimate final predictors of the individual biomarker variability and to examine the influence of confounding variables, multivariate analysis with stepwise regression was used. Only variables that were statistically different among Se tertiles were included in the model to estimate their contribution to the variability in the IsoP levels. Final models were analyzed to verify if the assumptions of linear regression were met. Colinearity between variables included in the final models was also assessed to avoid two variables highly correlated in the same model. For the stepwise regression, an α-value of 0.05 was used to exclude variables that had little or no influence on the biomarker under analysis. Sensitivity analyses were performed in order to assess whether results were similar after exclusion of individuals with subjects presented with acute inflammation (CRP ≥ 10 mg/l), diabetes, hypertension, stroke, or other CVD. We also verified whether exclusion of individuals taking medications for hypertension changed the regression coefficients. All statistical analyses were performed using SPSS version 13.0 software (SPSS Inc., Chicago, IL).

## RESULTS

A total of 294 subjects aged 18 years and older were randomly chosen from the IPY Inuit Health Survey from which specimens were available for laboratory analysis; and comprised the same subsample used in our previous study discussing plasma IsoPs in relation to CVD risk factors in more detail (27). According to AHA recommendations (32), CRP levels of 10 mg/l or greater represent evidence of active infection, systematic inflammatory processes, or trauma, and thus those individuals were excluded. A total of 294 subjects were assessed, of whom 60 subjects had CRP ≥ 10 and were excluded; in addition, one outlier with an F_2_-IsoP level below the detection limit was excluded, which decreased the sample size to 233 subjects. Complete ascertainment of the majority of variables was achieved; however there were missing data for F_2_-IsoPs (n = 2), IsoFs (n = 2), BMI (n = 8), WC (n = 11), blood Se (n = 6), blood Hg (n = 6), toenail Se (n = 38), SBP (n = 20), and LDL-C (n = 5). All data presented represent individuals with CRP < 10 mg/l. The mean age ± SD of the participants was 42.6 ± 15.4 years. The levels of F_2_-IsoPs and IsoFs and their ratio (IsoFs:F_2_-IsoPs) were within the normal range, as previously reported (**Table 1**) (27). Sixty-six subjects (28.3%) of our population subsample had plasma F_2_-IsoP levels ≥35 pg/ml, which is considered to be the upper value in the range for normal human plasma levels (30 ± 5 pg/ml) (33). Of the 233 participants (56% women), mean BMI was 27.78 kg/m^2^; 33.5% were overweight, and 30.4% were obese (Table 1). Based on self-reported medical histories, 5.7% of participants had diabetes, 28.6% had hypertension, 13.7% had dyslipidemia, 8.5% had cancer, 5.3% had a history of myocardial infarction, and 3.1% had a stroke. Seventy percent of participants were current smokers with 41.8% of smokers reporting >10 cigarettes/day; 65% of all participants reported drinking alcohol in the past year.

**TABLE 1.** Characteristics of study population (n = 233)

	Mean (SEM or 95% CI)
Age, years	42.56 (0.63)
Males/females, n	103/130
BMI	27.78 (0.38)
WC, cm	92.43 (0.97)
Body fat, %	29.63 (0.71)
SBP, mm Hg	117.36 (1.13)
DBP, mm Hg	76.54 (0.73)
Total cholesterol, mmol/l	5.02 (0.08)
LDL-C, mmol/l	2.92 (0.07)
HDL-C, mmol/l	1.47 (0.03)
Triglycerides, mmol/l	1.44 (0.72)
FG, mmol/l	4.97 (0.04)
CRP, mmol/l	1.66 (0.02)
Se, µg/l*	302.69 (281.84–324.41)
Toenail Se, µg/g	0.99 (0.02)
Hg, µg/l*	18.15 (15.79–20.82)
Hg:Se	0.50 (0.13)
F_2_-IsoPs, pg/ml*	27.35 (25.73–28.64)
IsoFs, pg/ml*	20.81 (18.86–22.96)
IsoF:F_2_-IsoP	0.92 (0.89–0.95)
Males/females (% females)	103/130 (55.8)
Current smoking, % yes	69.7
Alcohol consumption, % yes	55.8
Medication intake any,** % yes	38.4
Nutritional supplement any,^#^ % yes	9.6

DBP, diastolic blood pressure; FG, fasting glucose.

* Geometric mean (95% CI).

** Medications include self-reported prescription medications for diabetes, heart disease, dyslipidemia, cancer, and/or any others.

# Multivitamins, various B vitamins, salmon oil, cod liver oil, vitamin D, calcium, and Ensure.

### IsoPs, IsoFs, Se, and Hg

Blood and toenail Se were not correlated (*r* = −0.002, *P* = 0.98). F_2_-IsoPs were negatively correlated with both blood Se (*r* = −0.186, *P* < 0.01) and toenail Se (*r* = −0.146, *P* = 0.04) (**Table 2**). F_2_-IsoPs were not associated with either blood Hg (*r* = 0.057, *P* = 0.397) or Hg:Se (*r* = 0.109, *P* = 0.104). IsoFs were negatively associated with blood Se (*r* = −0.164, *P* = 0.014) but were not associated with toenail Se (*r* = −0.070, *P* = 0.335). IsoFs, however, were positively associated with blood Hg (*r* = 0.288, *P* < 0.001) and with Hg:Se (*r* = 0.340, *P* < 0.001). The IsoF:F_2_-IsoP ratio was not associated with either blood Se (*r* = −0.070, *P* = 0.301) or toenail Se (*r* = 0.014, *P* = 0.852); however, the IsoF:F_2_-IsoP ratio was positively associated with Hg (*r* = 0.269, *P* < 0.001) and with Hg:Se (*r* = 0.294, *P* < 0.001).

**TABLE 2.** Bivariate correlations between plasma IsoPs, tissue Se and Hg, and CRP

Pearson correlations	F_2_-IsoPs	IsoFs	IsoFs:F_2_-IsoPs
Blood Se			
*r*	−0.186**	−0.164*	−0.070
*p*	0.005	0.014	0.301
Toenail Se			
*r*	−0.146*	−0.070	0.014
*p*	0.044	0.335	0.852
Blood Hg			
*r*	0.057	0.288**	0.269**
*p*	0.397	<0.001	<0.001
Hg:Se			
*r*	0.109	0.340**	0.294**
*p*	0.104	<0.001	<0.001
CRP			
*r*	0.138*	0.158	0.074
*p*	0.037	0.016	0.262

* Significant at the 0.05 level.

** Significant at the 0.01 level.

### Comparison between Se tertiles

Participants were grouped into tertiles according to the blood Se distribution of the entire sample (<200, ≥200 and <340, and ≥340 µg/l) (**Table 3**). Blood Hg concentrations by Se tertiles showed wide and overlapping CIs, but the overall test for trend was significant with the highest Hg values noted in the highest Se tertile category. Plasma concentrations of both F_2_-IsoPs and IsoFs decreased with increasing tertiles of Se in unadjusted (Table 3) and adjusted analyses (**Table 4**).

**TABLE 3.** Characteristics of 233 subjects according to tertiles of blood Se concentrations

	T1 <200, Mean (95% CI)	T2 ≥200 and <340, Mean (95% CI)	T3 ≥340, Mean (95% CI)	*P* Trend^#^ Overall
Number of subjects	76	78	79	
Blood Se, µg/l	185.97^a^ (181.46–190.48)	258.18^b^* (250.63–265.73)	609.87^b^**^,c^* (550.14–669.60)	<0.01
Toenail Se, µg/g	0.97 (0.86–1.08)	1.00 (0.93–1.07)	0.99 (0.94–01.05)	0.83
Blood Hg, µg/l	29.32^ab^ (21.87–36.72)	22.44^a^ (15.92–28.96)	35.47^b^* (27.62–40.37)	0.026*
Hg:Se	0.53^a^ (0.47–0.59)	0.45^b^ (0.41–0.50)	0.52^ab^ (0.49–0.54)	0.037*
F_2_-IsoPs, pg/ml	29.64^a^ (26.75–32.85)	27.54^ab^ (25.28–30.22)	24.11^b^** (22.07–26.34)	<0.01**
IsoFs, pg/ml	28.98^a^ (24.46–34.33)	17.53^b^** (14.90–20.63)	18.14^b^** (15.24–21.60)	<0.01**
IsoFs:F_2_-IsoPs	1.00^a^ (0.95–1.05)	0.86^b^** (0.82–0.91)	0.91^b^* (0.86–0.97)	<0.01**
Age, years	39.28^a^ (35.90–42.66)	38.25^a^ (35.11–41.38)	49.92^b^** (46.44–53.41)	<0.01**
BMI, kg/m^2^	28.37 (26.91–29.84)	26.90 (25.65–28.15)	28.07 (26.82–29.33)	0.25
WC, cm	94.22 (90.65–97.80)	90.07 (86.93–93.21)	93.33 (89.84–96.81)	0.20
Body fat, %	30.94 (28.54–33.34)	27.87 (25.38–30.37)	30.04 (27.54–32.55)	0.19
SBP, mm Hg	116.25 (111.90–121.13)	115.86 (112.52–119.21)	119.52 (115.44–123.59)	0.35
DBP, mm Hg	76.52 (73.69–79.35)	76.85 (74.54–79.17)	76.29 (73.74–78.85)	0.94
Total cholesterol, mmol/l	4.73^a^ (4.48–4.98)	4.94^ab^ (4.71–5.17)	5.36^b^** (5.07–5.66)	<0.01**
LDL-C, mmol/l	2.63^a^ (2.42–2.85)	2.86^ab^ (2.66–3.07)	3.18^b^** (2.92–3.43)	<0.01**
HDL-C, mmol/l	1.44 (1.34–1.54)	1.47 (1.36–1.58)	1.53 (2.92–3.43)	0.48
Triglycerides, mmol/l	1.44 (1.18–1.70)	1.34 (1.18–1.50)	1.50 (1.18–1.81)	0.68
FG, mmol/l	4.88 (4.74–5.03)	4.90 (4.73–5.05)	5.12 (4.96–5.27)	0.049*
CRP, mmol/l	1.83 (1.46–2.26)	1.42 (1.14–1.74)	1.77 (1.43–2.18)	0.19

Missing data for few variables including F_2_-IsoPs (n = 2), IsoFs (n = 2), BMI (n = 8), WC (n = 11), blood Se (n = 6), blood Hg (n = 6), toenail Se (n = 38), SBP (n = 20), and LDL (n = 5) affected the final number of subjects for each tertile, maximum number of subjects reported. Unmatched superscript letters in the same row indicate significant statistical difference as determined by one-way ANOVA with Bonferroni adjusted multiple comparisons; whereby the mean difference is significant at the 0.05 level. T1, tertile 1; T2, tertile 2; T3, tertile 3; DBP, diastolic blood pressure; FG, fasting glucose.

*Significant at the 0.05 level.

**Significant at the 0.01 level.

#Trend at *P* > 0.05 and *P* ≤ 0.07.

**TABLE 4.** Adjusted mean and 95% CIs of plasma IsoPs by blood Se tertiles from general linear models

	T1 Se <200, Mean (95% CI)	T2 Se ≥200 and <340, Mean (95% CI)	T3 Se ≥340, Mean (95% CI)	F-test, Effect of Tertiles, F (*P* value)
Adjusted for age, sex, WC, and current smoking				
Number of subjects	76	78	79	—
F_2_-IsoPs, pg/ml	29.11^a^ (26.30–32.21)	28.31^ab^ (25.76–31.19)	24.21^b^* (21.93–26.73)	3.538 (0.035)
IsoFs, pg/ml	27.54^a^ (22.86–33.27)	19.14^b^* (16.07–22.80)	17.18^b^** (14.35–20.56)	6.890 (0.001)
IsoFs:F_2_-IsoPs	0.99^a^ (0.94–1.05)	0.88^b^* (0.83–0.94)	0.90^ab^ (0.84–0.95)	4.416 (0.013)
Adjusted for age, sex, WC, current smoking, LDL, FG, and Hg				
Number of subjects	76	78	79	—
F_2_-IsoPs, pg/ml	29.04^a^ (26.24–32.21)	28.91^ab^ (26.24–31.77)	24.10^b^* (21.82–26.55)	4.12 (0.018)
IsoFs, pg/ml	26.98^a^ (22.59–32.14)	19.82^b^* (16.83–23.39)	17.14^b^** (14.45–20.28)	6.678 (0.002)
IsoFs:F_2_-IsoPs	0.99^a^ (0.93–1.04)	0.89^b^* (0.84–0.94)	0.90^ab^ (0.84–0.95)	3.65 (0.028)

Missing data for F_2_-IsoPs (n = 2), IsoFs (n = 2), BMI (n = 8), WC (n = 11), blood Se (n = 6), blood Hg (n = 6), toenail-Se (n = 38), SBP (n = 20), and LDL-C (n = 5) affected the final number of subjects for each tertile, maximum number of subjects reported. Unmatched superscript letters in the same row indicate significant statistical difference as determined by one-way ANOVA with Bonferroni adjusted multiple comparisons; whereby the mean difference is significant at 0.05 level. T1, tertile 1; T2, tertile 2; T3, tertile 3; FG, fasting glucose.

*Significant at the 0.05 level.

**Significant at the 0.01 level.

### Final variance predictors of plasma IsoPs

Multivariate analyses (**Table 5**) indicated that F_2_-IsoP concentrations were significantly predicted by blood Se (β = −0.138, SE = 0.05, *P* = 0.01) and toenail Se (β =−0.096, SE = 0.04, *P* = 0.026), in separate analyses of these Se biomarkers adjusted for WC (β = 0.003, SE = 0.001, *P* < 0.001) and gender (β = 0.052, SE = 0.63, *P* = 0.038). F_2_-IsoPs were not associated with Hg but were associated with the ratio of Hg:Se (β = 0.148, SE = 0.07, *P* = 0.021). Variance in IsoF concentrations was predicted by both Se (β = −0.238, SE = 0.02, *P* = 0.016) and Hg (β = 0.222, SE = 0.05, *P* < 0.001) and Hg:Se (β = 0.534, SE = 0.11, *P* < 0.001) in addition to WC (β = 0.007, SE = 0.002, *P* < 0.001) and age (β = −0.003, SE = 0.002, *P* = 0.042). Variance in the IsoF:F_2_-IsoP ratio was predicted by Hg (β = 0.118, SE = 0.03, *P* < 0.001), Hg:Se (β = 0.323, SE = 0.08, *P* < 0.001), and WC (β = 0.003, SE = 0.001, *P* = 0.014).

**TABLE 5.** Multivariate associations showing the regression coefficient (β) of plasma IsoP concentrations

	Stepwise Regression	F_2_-IsoPs β (SE)	IsoFs β (SE)	IsoF:F_2_-IsoPs β (SE)
Model (a)	Constant	1.449 (0.16)	1.264 (0.28)	0.664 (0.10)
	Independent variables:			
	Se	−0.138 (0.05)*	−0.238 (0.02)*	NS
	WC	0.003 (0.001)**	0.007 (0.002)**	0.003 (0.001)**
	Sex	0.052 (0.63)**	—	—
	R^2^	0.104	0.121	0.038
	F	7.13**	12.75**	7.31**
Model (b)	Constant	1.16 (0.09)	0.55 (0.15)	0.528 (0.11)
	Independent variables:			
	Mercury	NS	0.222 (0.050)**	0.118 (0.03)**
	WC	0.003 (0.001)**	0.007 (0.002)**	0.003 (0.03)*
	Sex	0.054 (0.03)*		
	Age	−0.002 (0.001)**	−0.003 (0.002)*	—
	R^2^	0.101	0.182	0.102
	F	6.90**	13.73**	10.56**
Model (c)	Constant	1.25 (0.10)	0.68 (0.15)	0.64 (0.111)
	Independent variables:			
	Toenail Se	−0.096 (0.04)*	NS	NS
	WC	0.003 (0.001)**	0.007 (0.002)**	0.003 (0.001)*
	Sex	0.059 (0.03)*	—	—
	Age	−0.002 (0.001)*	—	—
	R^2^	0.13	0.09	0.04
	F	5.99**	17.09**	6.53*
Model (d)	Constant	1.12 (0.09)	0.46 (0.15)	0.52 (0.11)
	Independent variables:			
	Hg:Se	0.148 (0.07)*	0.534 (0.11)**	0.323 (0.08)**
	WC	0.003 (0.001)**	0.006 (0.002)**	0.003 (0.001)*
	Sex	0.053 (0.07)*	—	—
	Age	−0.003 (0.001)**	—	—
	R^2^	0.12	0.19	0.12
	F	6.51**	22.22**	12.14**

In all models independent variables included: WC, sex, age, smoking, alcohol, diabetes mellitus or fasting glucose, hypertension or SBP or CRP, hyperlipidemia, or total cholesterol on a continuous scale. NS, not significant.

*Significant at the 0.05 level.

**Significant at the 0.01 level.

## DISCUSSION

This study demonstrates that Inuit, who represent a unique population with elevated blood concentrations of both Hg and Se, showed variations in plasma IsoPs in relationship to both tissue Se and Hg. Despite high Hg exposure exceeding levels reported in Caucasians (34, 35), the mean plasma concentration of F_2_-IsoPs for the population as a whole was within the normal range for healthy humans (33) and just over a quarter of the sample had levels exceeding the upper range value (35 pg/ml). In concordance with previous reports regarding Inuit of Nunavik (17), our findings confirm that Hg presence in the traditional diet may not be of major concern with respect to oxidative stress. The observation of a positive association of Hg with F_2_-IsoPs only when tissue Hg was considered in the form of the Hg:Se ratio is novel because human studies have not previously evaluated the interaction of tissue Hg and Se on plasma IsoPs. A lack of relationship between Hg and plasma F_2_-IsoPs and other lipid peroxidation biomarkers has been previously reported in a group of premenopausal women with low blood Hg concentrations (mean of 1.10 µg/l; interquartile range of 0.58–2.0), but tissue Se was not evaluated (36). In further support of the relationship of Hg and Se on oxidative stress, we observed that Inuit in the two highest tertiles of blood Se were most protected from oxidative stress independent of other covariates that could influence Se status including tissue Hg, age, cardiometabolic risk factors, smoking, and blood lipids.

To our knowledge, this is the first human study to demonstrate protective effects of tissue Se status on oxidative stress status in relation to MeHg exposure. The above findings corroborate the findings of experimental animal studies where the presence of both Se and Hg within the same food matrix minimizes Hg-induced oxidative stress (13). The modulation of Hg-mediated induction of F_2_-IsoPs by tissue Se is also consistent with recent observations that Se supplementation prevented the increase in urinary F_2_-IsoPs induced by dietary MeHg exposure in rats (37). MeHg has repeatedly and consistently been shown to be an irreversible inhibitor of selenoenzymes (14, 15), which can be counteracted by higher Se intakes that provide added free Se to support Se-dependent selenoprotein synthesis (38). Additionally, Se can provide protection by affecting the kinetics and metabolism of Hg. In one small randomized trial, Seppanen et al. (39) found that Se supplementation in a Finnish cohort with low Se intakes reduced hair Hg levels by one third over four months. Li et al. (40) found that when five volunteers from the Wanshan mercury mining area were supplemented with Se-enriched yeast for three months, an increased urinary Hg excretion was observed.

Although both IsoFs and F_2_-IsoPs were positively associated with the tissue Hg:Se ratio in our study, only IsoFs and the IsoF:F_2_-IsoP ratio were positively associated with tissue Hg content alone. The latter finding could be explainable by observations that the ratio of IsoFs to F_2_-IsoPs reflects ambient oxygen concentrations within the environment in which lipid peroxidation occurs (26). Thus, as ambient oxygen concentrations increase as a result of mitochondrial disruption, the ratio is skewed toward IsoF formation and away from F_2_-IsoP production (26). In that regard, impaired mitochondrial oxygen consumption has been noted upon Hg exposure (41), which has been related to altered structural integrity of the mitochondrial inner membrane that is highly sensitive to oxidative stress (42). Interestingly, Inuit from Nunavik and sports fisherman from James Bay in Québec exposed to high dietary Hg showed a higher ubiquinone-10 to ubiquinone-10-CoQ_10_ total redox ratio (17, 43), which could reflect disrupted mitochondrial activity of thioredoxin that regenerates important antioxidants including ubiquinone (44). Further studies are needed to evaluate the utility of IsoFs as a sensitive biomarker of Hg exposure's systemic effects.

Recent concerns have been raised that the high Hg content found in Northern traditional food may contribute to the development of cardiometabolic disorders (19, 45). We have previously demonstrated that oxidative stress in the Inuit is related to obesity-induced inflammation and F_2_-IsoPs and IsoFs were both related significantly to SBP and CRP (27). In our current analyses, however, SBP was not retained in the stepwise regression analyses evaluating associates of IsoPs where Hg, Se, toenail Se, or the Hg:Se ratio were significant along with age, WC, and sex. The above results imply that the relationship of F_2_-IsoPs and IsoFs with either CRP or SBP in the Inuit population is Se and Hg codependent and is also suggestive of protective effects of Se on cardiometabolic disturbances associated with IsoPs. We speculate that the latter observation is related to the antioxidative and anti-inflammatory properties of the different GSH peroxidases (46) that could be induced by the high tissue content of Se seen in this study. Further, Se may inhibit the activation of nuclear factor-κB by modulation of selenoprotein gene expression (47) that, in turn, impedes the transactivation of genes that encode pro-inflammatory cytokines (48). In addition, dietary Se may inhibit the biotransformation of arachidonic acid toward the formation of prostaglandins and thromboxanes that promote inflammation (49). Our results differ from Valera, Dewailly, and Poirer (50) who showed a significant positive correlation between blood Hg concentrations and both SBP and pulse pressure in Inuit adults from Nunavik, after considering the confounding effects Se, n-3 PUFAs, and other correlated variables. Our population had higher mean blood Hg concentrations compared with the cohort in Nunavik [90.75 (78.95–114.8) nmol/l vs. 20.2 (46.6–54.1) nmol/l] with comparable Se and n-3 PUFA concentrations, which should have allowed us to detect any adverse effect of Hg in our cohort.

Unlike the Nunavik studies (17, 50), Hg and Se were not highly correlated (*r* = 0.323, *P* < 0.001), which could indicate regional differences in the type of traditional food consumed or variability in Se and Hg content of traditional food consumed. Factors shown to affect F_2_-IsoPs such as n-3 PUFA (51) did not correlate with either of the plasma IsoPs measured in this study and thus was not included in further analyses. While most Hg exposure among Inuit is likely to be due to intake of MeHg from traditional foods, we did not separately examine different forms of Hg (inorganic, methyl) which may exert different effects on oxidative stress biomarkers. In addition, many other dietary factors that can further explain F_2_-IsoP variability were not included in this study, including plasma antioxidants such as α-tocopherol, ascorbic acid, polyphenols, and carotenoids. The nonlinear associations between Hg and Se across Se tertiles could be attributable to residual confounding resulting from not including other important constituents of the diet. Seasonal variability in exposures and associations could not be assessed.

To our knowledge, this is the first human study to report a relationship between tissue Hg and plasma F_2_-IsoP concentrations. Our finding that oxidative stress associated with Hg exposure was highly modulated by tissue Se status emphasizes the importance of concurrent tissue Se measurement for the assessment of Hg-mediated oxidative stress. We have also shown that concurrent measurement of Hg and Se can provide new insights with respect to quantifying risk of oxidative stress as a mechanistic link to the progression to CVD. More studies are needed to confirm our observations using larger sample sizes in order to perform more complex analyses dealing with Hg and Se interactions.
